# Keratometric measurements and IOL calculations in pseudophakic post-DSAEK patients

**DOI:** 10.1186/s12886-018-0931-y

**Published:** 2018-10-17

**Authors:** Ke Xu, Hong Qi, Rongmei Peng, Gege Xiao, Jing Hong, Yansheng Hao, Boping Ma

**Affiliations:** Department of Ophthalmology, Beijing Key Laboratory of Restoration of Damaged Ocular Nerve, Peking University Third Hospital, 49 North Garden Road, Haidian District Beijing, 100191 People’s Republic of China

**Keywords:** Descemet’s stripping automated endothelial keratoplasty, IOL, Keratometry, Pentacam

## Abstract

**Background:**

To compare different K readings in pseudophakic patients post-Descemet’s stripping automated endothelial keratoplasty **(**DSAEK) and evaluate corresponding prediction errors in intraocular lens (IOL) power calculations.

**Methods:**

Subjects that underwent cataract surgery and DSAEK surgery at least 3 and 6 months prior, respectively, and IOL implantation in the capsular bag were included in this study. Manifest refraction and IOL information were recorded. A Scheimpflug keratometer (Pentacam) was used for corneal measurements, including the mean anterior and posterior radii of curvature, simulated keratometer (SimK), true net power (TNP), and equivalent K reading (EKR) at the 4.0-mm zone. Conventional keratometry was acquired using the IOLMaster (K_Master_). The four K measurements were evaluated for calculating the predicted refraction.

**Results:**

The study included 20 eyes from 19 subjects. The ratio of the posterior to the anterior corneal radius was 74.1 ± 3.24%. Comparison of the four keratometric methods (K_Master_, SimK, EKR, and TNP) revealed statistically significant differences among all the methods besides K_Master_ and SimK. Of the four IOL calculation methods(K_Master_, SimK, EKR and TNP method),the arithmetic prediction error of the K_Master_, SimK, and EKR methods featured nonsignificant differences from zero(*p* = 0.07, 0.19 and 0.84 respectively); the EKR method calculated the highest percentage of eyes with IOLs within the prediction error.

**Conclusions:**

IOL calculations in post-DSAEK eyes using K_Master_, SimK, and EKR can yield small refractive errors after surgery. The EKR (4.0-mm diameter) method was found to be the most accurate.

## Background

Accurate assessment of the total corneal power of eyes following corneal refractive surgery is essential for determining optimal intraocular lens (IOL) power, and the difficulties in accurately evaluating corneal power after laser-assisted in situ keratomileusis (LASIK) have been well described [1, 2]. Descemet’s stripping automated endothelial keratoplasty (DSAEK) procedure is a lamellar corneal surgical technique used to replace the abnormal corneal endothelium of patients with endothelial disease [3]. The literature features a dearth concerning the proportion of DSAEK performed on phakic eyes without concomitant cataract surgery relative to the total number of DSAEK conducted. The technical advantages of DSAEK encouraged surgical intervention earlier in the course of clinical treatment, resulting in an increased number of patients presenting with clear lenses at the time of corneal intervention [4, 5]. This is particularly the case for patients with familial Fuchs dystrophy and clinically significant corneal disease that requires intervention at a relatively early age [6]. When these patients develop a clinically significant cataract post-DSAEK, changes on the posterior surface of the cornea affect the accuracy of keratometry (K) measurements and subsequent IOL power calculations. Previous studies have compared corneal power parameters after DSAEK with those of control or pre-DSAEK groups [7–11]; however, data on the accuracy of K measurements in post-DSAEK corneas and the prediction error in IOL power calculations among post-DSAEK patients is lacking.

The present retrospective study therefore aimed to compare K readings obtained with a conventional keratometer (IOLMaster) and a Scheimpflug keratometer in pseudophakic, post-DSAEK patients to evaluate prediction errors in IOL power calculations.

## Methods

The present study conducted a retrospective review of 19 pseudophakic patients who underwent DSAEK at Peking University Third Hospital’s Department of Ophthalmology and whose initial visit occurred between February 2016 and July 2016. The investigation was performed according to the tenets of the Declaration of Helsinki. The need for informed consent was waived and the study protocol was approved by Peking University Third Hospital Medical Science Research Ethics Committee.

Exclusion criteria included the following: history of ocular trauma, scarring, or severe edema of the corneal stroma; capsular or zonular anomalies of the lens; silicone oil in the posterior segment; diabetic macular changes; pathologic myopia; best-corrected visual acuity (BCVA) of < 20/100; and the inability to complete post-operative examinations. The present study included subjects whose cataract and DSAEK surgery needed to have been completed at least 3 and 6 months prior, respectively.

All patients underwent a routine phacoemulsification procedure and implantation of a foldable IOL in the capsular bag. Cataract surgery could occur prior to, in conjunction with, or after the performance of DSAEK, which were all performed by a single surgeon (J. Hong) according to a previously described procedure [12, 13]. The cataract surgery date, DSAEK surgery date, IOL type, and diopter information were recorded.

Patients attended regularly scheduled examinations that included evaluations of manifest refraction, uncorrected visual acuity, and BCVA; a slit-lamp examination; and anterior segment optical coherence tomography (Visante Model 1000, Carl Zeiss Meditec) to obtain total cornea and graft thickness(CT and GT,respectively) at the vertex and 2-mm points(0°, 90°, 180°, 270°). The Pentacam rotating Scheimpflug imaging system (Oculus, Wetzlar, Germany) was used for all corneal measurements: mean anterior radius of curvature, mean posterior radius of curvature, simulated keratometer reading (SimK), true net power (TNP), and equivalent K reading (EKR) at the 4.0-mm zone. The scans were repeated if the device did not issue a quality output reading of “OK.” Only patients with a quality output reading of “OK” were included in the study. Conventional K and axial length (AL) measurements were acquired by the IOLMaster (Carl Zeiss, Meditec AG, Jena, Germany) using the default settings for pseudophakic eyes.

A simplified relationship between the K readings (in diopters) and the value of the anterior corneal radius r (in millimeters) was used to calculate conventional K acquired by the IOLMaster(K_Master_): K_Master_ = 0.3375/r_anterior_. The principle of SimK from the Pentacam system used in this study is identical to the conventional keratometric method: SimK = 0.3375/ r_anterior_.TNP represents the sum of the anterior and posterior corneal powers [14]. This value was calculated using the following formula (1):TNP = 0.376/r_anterior_ − 0.04/r_posterior_. In untreated eyes, r_posterior_ can be substituted by 0.822r_anterior_ [15, 16]:TNP_untreated_ = 0.3273/r_anterior_. EKR was advanced by Holladay and his colleagues to evaluate the total corneal power after corneal refractive surgery and was calculated using the following formula(2): EKR = 0.376/ r_anterior_– 0.3165/ r_posterior_ [17]. In untreated eyes, r_posterior_ can be substituted by 0.822r_anterior_ [15, 16]:EKR_untreated_ = 0.337496/ r_anterior._

The different K readings were introduced into the calculation formula attached to the IOLMaster to obtain the predicted refraction. The IOL power calculations were performed using third-generation formulas (SRK/T for AL > 26 mm, Hoffer Q for AL < 22 mm, and Holladay 1 for AL from 22 to 26 mm) as recommended by Hoffer with optimized A-constants for different implanted IOLs [18]. Predicted refraction was collected from the biometry reading of the selected IOLs. Achieved refraction was the spheroequivalent value of the manifest refraction. The arithmetic prediction error (achieved refraction minus predicted refraction), absolute error, and percentage of eyes within 0.5 D, 1.0 D, and 2.0 D were calculated. According to Chang [19], an adjustment of target refraction by 0.17 to 0.24D is required to simulate a phakic eye model. This range was calculated as the approximate mean of the hyperopic shift between the pseudophakic predicted refraction and the phakic predicted refraction. Therefore, all prediction refraction values were adjusted by a decrease of 0.2 D, the mean of 0.17 and 0.24 D.

Statistical analyses were performed using SPSS for Windows (version 16.0, SPSS, Inc.). A Student’s t-test was used to evaluate the presence of significant differences in the data. The relationship between the differences in K readings and the corneal profiles was assessed via regression analysis. A *p-*value of < 0.05 was considered statistically significant.

## Results

The present study evaluated 20 pseudophakic eyes (right, 12; left, 8) from 19 patients (five men) who had undergone DSAEK. The mean age of the patients was 70 ± 12 years (range, 38–83 years). Prior to DSAEK surgery, the cohort included six eyes with Fuch’s endothelial dystrophy, six eyes with corneal endothelial decompensation of unknown etiology, and eight eyes with post-cataract or post-IOP-elevation bullous keratoplasty. Of the 20 eyes, one underwent DSAEK prior to cataract surgery, seven received DSAEK after cataract surgery, and 12 underwent the two surgeries during the same procedure. The mean postoperative times following the cataract and DSAEK surgeries were 730 days (range 183–1524 days) and 626 days (range 181–1402 days), respectively. The mean spheroequivalent value of achieved refraction was − 1.43 D (range − 6.25 to 2.13 D). The mean AL was 24.09 ± 2.87 mm (range 21.08 to 31.50 mm). AL of < 22.0 mm (Hoffer Q formula) featured in two eyes (10%); 15 eyes (75%) had an AL of 22.0–26.0 mm (Holladay 1 formula); and three eyes (15%) had an AL of > 26.0 mm (SRK/T formula). All 20 implanted IOLs were acrylic and placed in the capsular bag. The models of the IOLs were as follows: nine were Model 400, Medennium, Inc.; five, AR40e, Abbott Medical Optics, Inc.; three, YA-60BB, Hoya Corporation; one, ZCB00, Abbott Medical Optics, Inc.; one, HQ-201HEP, HexaVision SARL; and one, ZA9003, Abbott Medical Optics, Inc. The mean anterior corneal radius was 7.68 ± 0.30 mm, and that of the posterior corneal radius was 5.69 ± 0.28 mm; the ratio of the posterior to the anterior corneal radius was 74.1 ± 3.24% (range 67.8–81.8%). The mean central graft thickness was 117.25 ± 41.83 μm. The mean graft thickness at the 2-mm corneal point was 138.74 ± 46.05 μm.

**Fig. 1:**
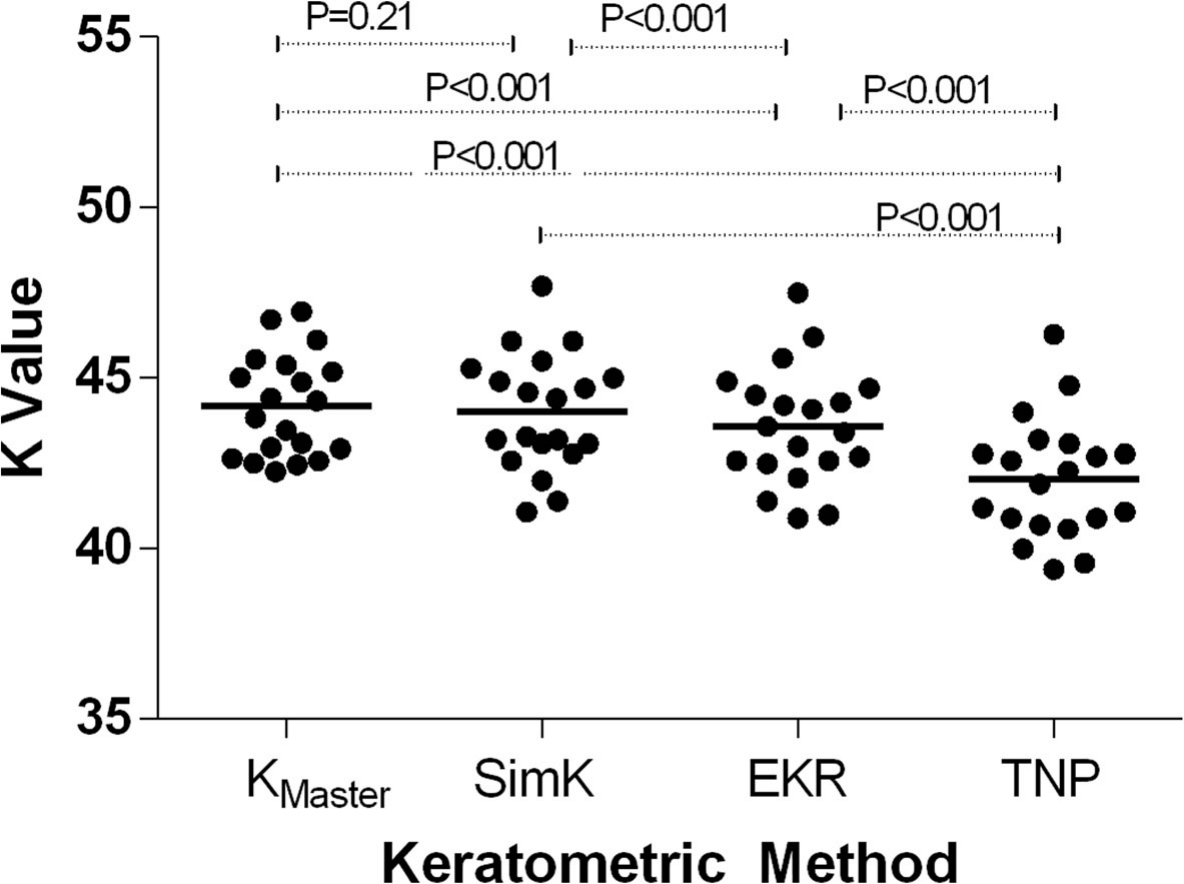
Scatterplot of mean K values for the keratometric methods. Statistically significant differences were observed in all t-test pairs, except between K_Master_ and SimK. K_Master_, conventional K obtained with the IOLMaster; SimK, simulated keratometer reading; EKR, equivalent K reading; TNP, true net power

Figure 1 shows the K values for each keratometric method. Comparisons among the four keratometric methods [Conventional K from the IOLMaster (K_Master_) and SimK, EKR, and TNP from the Pentacam] revealed no statistically significant difference between K_Master_ and SimK; statistically significant differences were, however, detected among all other comparisons among the keratometric methods. K_Master_ (44.18 ± 1.50) and SimK (44.01 ± 1.71) featured the largest mean K-values, followed respectively by EKR (43.59 ± 1.72) and TNP (42.05 ± 1.77). Table 1 shows the differences in the observed K values in relation to TNP.

**Table 1:** Mean K values of different keratometric methods and comparison between those K values and TNP

				Difference from TNP		
Type of K	n	Mean(D) ± SD	Range	Mean (D) ± SD	95% CI of difference	*P* value
K_Master_	20	44.18 ± 1.50	42.28 to 46.95	2.13 ± 0.61	1.85 to 2.41	< 0.001
SimK	20	44.01 ± 1.71	41.10 to 47.70	1.96 ± 0.33	1.81 to 2.11	< 0.001
EKR at 4.0 mm	20	43.59 ± 1.72	40.90 to 47.50	1.55 ± 0.27	1.42 to 1.67	< 0.001
TNP	20	42.05 ± 1.77	39.40 to 46.30	–	–	–

*K*_*Master*_ conventional K via IOLMaster, *SimK* simulated keratometer reading, *EKR* equivalent K reading, *TNP* true net power

**Fig. 2:**
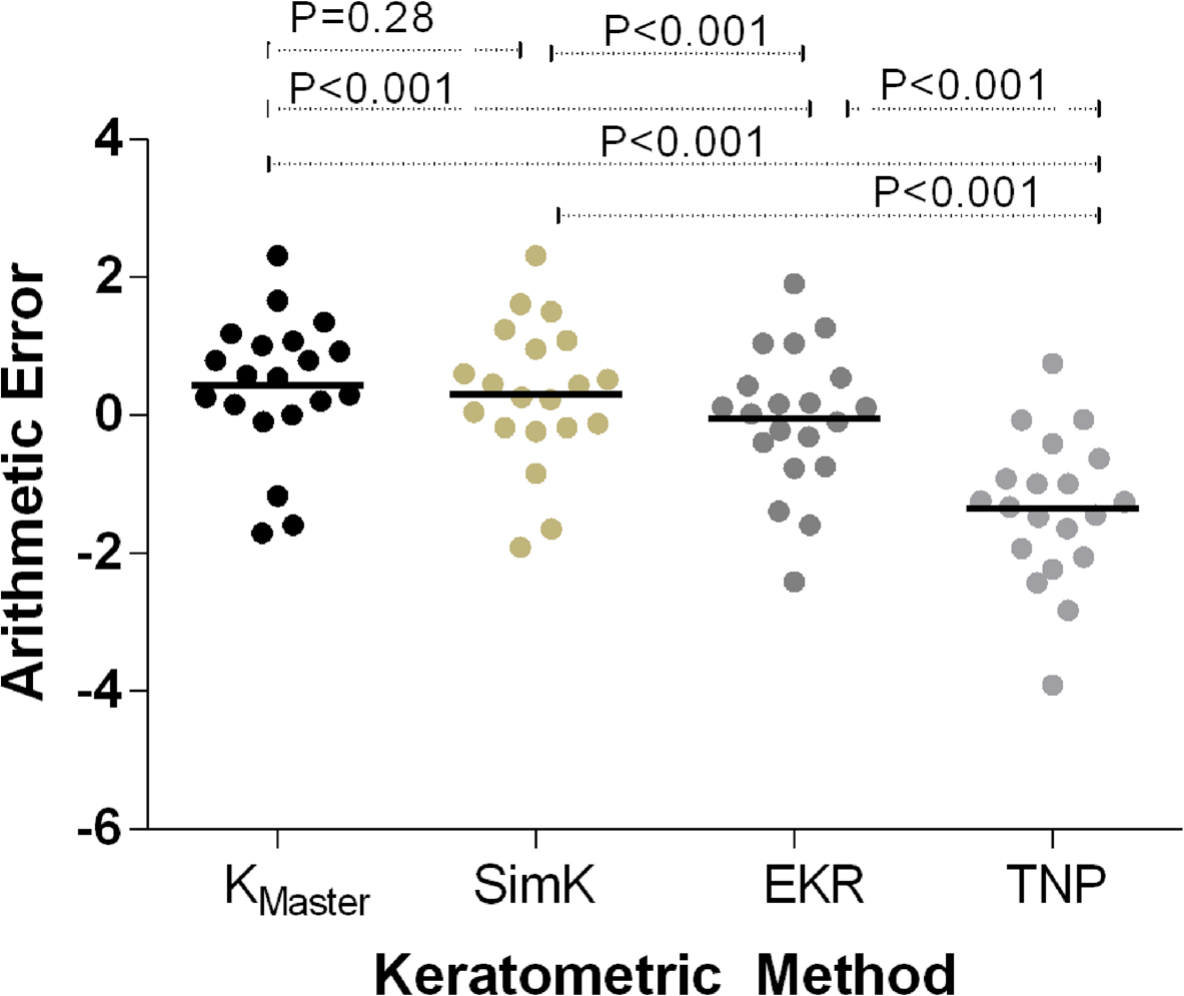
Arithmetic error plot using different K reading methods. K_Master_, conventional K obtained with the IOLMaster; SimK, simulated keratometer reading; EKR, equivalent K reading; TNP, true net power

Table 2 shows the relationship between the corneal profile and K reading differences. There was a very strong correlation between the ratio of the posterior to the anterior corneal radius and the SimK-TNP difference (r^2^ = 0.73, *p* < 0.001). Table 3 shows the arithmetic error and absolute error of predictions using the four K readings. Comparison of arithmetic errors using the different Ks and a paired t-test found no statistically significant difference between K_Master_ and SimK; statistically significant differences were found for all other comparisons among the keratometric methods (Fig. 2). A paired t-test revealed no statistically significant differences among the absolute errors of K_Master_, SimK, and EKR.

**Table 2:** Relationship between corneal profile and K reading differences

Difference of Method	Correlated factor	r^2^	*P* value
K_Master_ - TNP	R_posterior_/R_anterior_	0.19	0.05
	central/peripheral CT	0.24	0.03*
	central/peripheral GT	0.11	0.16
Sim K- TNP	R_posterior_/R_anterior_	0.73	< 0.001*
	central/peripheral CT	0.42	0.00*
	central/peripheral GT	0.29	0.01*
EKR at 4.0 mm - TNP	R_posterior_/R_anterior_	0.16	0.09
	central/peripheral CT	0.16	0.08
	central/peripheral GT	0.15	0.09

*K*_*Master*_ Conventional K via IOLMaster, *SimK* Simulated keratometer reading, *EKR* Equivalent K reading, *TNP* True net power, *R posterior* mean posterior radius of the corneal curvature, *R anterior* mean anterior radius of the corneal curvature, *CT* total corneal thickness, *GT* corneal graft thickness. *p < 0.05

**Table 3:** Arithmetic error and absolute error of prediction using different K readings

Method	n	Arithmetic Error (D)			Absolute Error (D)		
		Mean(D) ± SD	Minimum - Maximum	Range	Mean(D) ± SD	Minimum - Maximum	Range
K_Master_	20	0.44 ± 1.02	−1.70 to 2.32	4.02	0.89 ± 0.63	0.01 to 2.32	2.31
SimK	20	0.32 ± 1.03	−1.90 to2.32	4.22	0.82 ± 0.68	0.05 to 2.32	2.27
EKR	20	−0.05 ± 1.02	−2.40 to 1.91	4.31	0.74 ± 0.68	0.03 to 2.40	2.37
TNP	20	−1.35 ± 1.06	−3.90 to 0.76	4.66	1.42 ± 0.95	0.05 to 3.90	3.85

*K*_*Master*_ conventional K via IOLMaster, *SimK* simulated keratometer reading, *EKR* equivalent K reading, *TNP* true net power

Of the four IOL calculation methods [the IOLMaster-measured K and three Pentacam-measured corneal powers (SimK, EKR and TNP) inserted into the three third-generation formulas], only the arithmetic error of the TNP method was significantly different from zero (one-sample t-test, *p* < 0.01); that of the other three methods (K_Master_,SimK and EKR) featured nonsignificant differences with a value of zero (*p* = 0.07, 0.19, and 0.84, respectively). The percentages of eyes within 0.50, 1.00, and 2.00 D, and that exceeding 2.00 D of the prediction error of the three different methods are shown in Table 4. The EKR method featured the highest percentage values for each category and the lowest mean absolute prediction error value (0.74 ± 0.68).

**Table 4:** Percentage of eyes within 0.50 D, 1.00 D, and 2.00 D, and exceeding 2.00 D of the prediction error of the 3 different methods

Method	Prediction Error (%)			
	Within ±0.50D	Within ±1.00D	Within ±2.00D	> 2.00D
K_Master_	30	55	95	5
SimK	45	65	95	5
EKR	50	65	95	5

*K*_*Master*_ conventional K via IOLMaster, *SimK* simulated keratometer reading, *EKR* equivalent K reading

## Discussion

In the present study, we investigated four keratometric methods and predicted their respective post-DSAEK IOL-calculation error in pseudophakic patients. Several studies reported a hyperopic shift after DSAEK, ranging from 0.7 to 1.5 D [1, 3, 8, 9, 20, 21]. The hyperopic shift is partially explained by the meniscus-shaped configuration of the endothelial graft, which is thicker in the periphery and likely contributes to the increased curvature of the posterior surface [2, 9, 21, 22]. We found that after DSAEK, the normal physiologic relationship between the anterior and posterior surfaces, on which the conventional K measurement is based, was altered; the accuracy of the K measurement and subsequent IOL-power calculation was consequently affected. To the best of our knowledge, this is the first study to report IOL calculations in post-DSAEK patients.

The mean ratio of the posterior corneal radius to the anterior corneal radius of an untreated cornea is 82.2% [15, 16]. In post-DSAEK patients, we found that this ratio decreased to 74.1% (SD 3.24%), confirming that posterior lamellar grafts alter the corneal profile. When a non-uniform thickness graft, thicker in its periphery than in its center, is added to the posterior host cornea, it contributes to the reduction of the posterior corneal radius of curvature and thereby decreases the ratio of the posterior corneal radius to the anterior corneal radius. Conventional keratometry assumes that the radius of the curvature of the posterior ocular surface is 82.2% that of the anterior corneal surface. This explains the significant difference in the corneal powers detected by K_Master_ and TNP (44.18 vs. 42.05 D, *p* < 0.01), as well as by SimK and TNP (44.01 vs. 42.05 D, *p* < 0.01).

For subjects who underwent DSAEK, the ratio of the posterior to anterior corneal radii was 0.741. Hence, r_posterior_ can be substituted by 0.741r_anterior_ in formula1 and 2. The TNP and EKR for these subjects were calculated as follows: TNP_DSAEK_ = 0.3220/ r_anterior_;EKR_DSAEK_ = 0.3333/ r_anterior_. Table 5 shows the different K formulae used for the measurements obtained before and after the DSAEK; the different coefficients used in these formulae can partially account for the differences and sequence in keratometry. Previous studies on virgin eyes found that K_Master_ and SimK values were higher than those of TNP by approximately 1.13–1.43 D [23–27]. The present study observed a post-DSAEK difference of approximately 2.13 D between K_Master_ and TNP and a difference of 1.96 D between SimK and TNP; both differences are greater than those found in virgin eyes. While The coefficients in the formulae for K_Master_ and SimK did not change after DSAEK, the smaller coefficient of the TNP formula decreased after DSAEK. The latter finding may account for the change in K distance after DSAEK.

**Table 5:** Different K formulas before and after DSAEK surgery

Untreated Eye	After DSAEK
K_Master_ = 0.3375/r_anterior_	K_Master_ = 0.3375/r_anterior_
Sim K = 0.3375/r_anterior_	Sim K = 0.3375/r_anterior_
EKR_untreated_ = 0.337496/r_anterior_	EKR_DSAEK_ = 0.3333/r_anterior_
TNP_untreated_ = 0.3273/r_anterior_	TNP_DSAEK_ = 0.3220/r_anterior_

Accounting for both the anterior and posterior corneal surfaces, TNP may more accurately reflect the actual corneal refractive power than the other K values. Our regression analysis of the corneal profile and SimK-TNP difference revealed that the latter was strongly correlated with the ratio of the posterior to the anterior corneal radius (r^2^ = 0.73*, p* < 0.001). We further found that the difference between the SimK and TNP was weakly correlated with the central/peripheral CT (r^2^ = 0.42) and the GT (r^2^ = 0.29). The possible reason is that measuring the thickness 2 mm from the vertex of the total cornea and graft cannot reflect the peripheral profile and, when compared with the ratio of the posterior to the anterior corneal radius (mean radius on a 3-mm ring), the latter may better reflect the corneal profile.

Despite the fact that the same principle underlies the Pentacam (SimK) and IOLMaster (K_Master_) systems, the present study failed to observe a correlation between the keratometric power deviation (K_Master_ versus TNP) and the ratio of the posterior to the anterior corneal radius (r^2^ = 0.19*, p* > 0.05). This may be explained by the parameters of SimK, TNP, and the ratio of the posterior to anterior corneal radius been derived from the same corneal topography system, whereas that for K_Master_ is not. Our finding that the EKR-TNP difference was not correlated with the corneal profile may be accounted for by the following: the mean value of EKR was between those of SimK and TNP and the deviation from TNP was small.

Employing the original implanted IOL power and optical biometry, Chang et al. [19] reported that the IOLMaster predicted more hyperopic refraction in pseudophakic eyes and observed a mean hyperopic shift of approximately 0.17D to 0.24 D in the pseudophakic-predicted refraction relative to those calculated from phakia using the SRK II and SRK/T formulae. The subjects in the present study also exhibited a pseudophakic condition. To simulate the phakic eye, 0.20 D (the mean of 0.17D and 0.24 D) was subtracted from each predicted refraction to compensate for our use of third-generation IOL formulae. The arithmetic prediction errors of the IOL calculations using K_Master,_ SimK, and EKR were not significantly different from zero. Comparison of the arithmetic error of the three methods using a paired t-test revealed statistically significant differences between EKR and SimK as well as between EKR and K_Master_. The percentages of eyes within 0.50, 1.00, and 2.00 D, and that exceeding 2.00 D of the prediction error of the three different methods confirmed that the EKR method produced the highest percentage values for each category. Considering that the mean value of the absolute prediction error of the EKR method was the lowest (EKR, 0.74; SimK, 0.82; K_Master_, 0.89), we suggest that EKR be used in IOL calculations in post-DSAEK eyes. Benchmarks for refractive success after routine cataract surgery are reported as 85% within 1.0 D and 55% within 0.5 D of the intended refraction [28]. As 50% of patients were within 0.5 D of the predicted value, the present study demonstrates that an accuracy would be nearly achievable by using the 4.0-mm-diameter EKR.

Conventional keratometry has been noted to overestimate corneal power in patients after myopic laser refractive surgery, which may result in an undesirable, unexpectedly large hyperopic refractive error [29–31]. The present study found that the arithmetic prediction error of K_Master_ and SimK was not significantly different from zero, indicating that although LASIK and DSAEK both change the relationship between the anterior and posterior corneal refractive powers, the latter induces a smaller impact on corneal refractive power than does the former. This finding may be accounted for by the following mechanisms. (i) Unlike LASIK, changes in the corneal power after DSAEK mainly occur in the posterior cornea, which has low refractive power [9, 32]. (ii) Post-DSAEK eyes feature a graft diameter that is sufficiently large to maintain a continuous change in the central-area curvature, further allowing paracentral measurement of conventional keratometry to be conducted. (iii) Improvements in the preparation of DSAEK grafts reduce variations in the corneal profile, thus eliminating postoperative changes in refraction.

The present investigation was subject to the limitation of a small sample size and those inherent to retrospective studies. Pseudophakic post-DSAEK eyes were evaluated, but adjustments were required to simulate phakic post-DSAEK eyes. Using EKR in a clinical scenario would therefore likely achieve less than the 50% within 0.5 D of the intended refraction. Further, although some cases indicated that intraocular surgery with appropriate precautions could be performed safely in post-DSAEK eyes, K measurements may be affected by phacoemulsification-related damage to the corneal endothelium during cataract surgery post-DSAEK [33]. Future prospective clinical studies from multiple centers will help address and attenuate these limitations.

## Conclusions

The ratio of the posterior to the anterior corneal radius of curvature decreased to74.1% following DSAEK. The deviation between SimK versus TNP in post-DSAEK eyes increased and was strongly correlated with the ratio of the posterior to the anterior corneal radius of curvature. IOL calculations in post-DSAEK eyes using K_Master_, SimK, and EKR can yield small refractive errors after surgery; however, EKR (4.0-mm diameter) was found to be the most accurate.

WHAT WAS KNOWN

- Accurate assessment of the total corneal power in eyes after corneal refractive surgery is essential for determining the optimal IOL power. The difficulty in accurately evaluating corneal power after LASIK surgery is well established.
- In patients with cataracts post-DSAEK, changes in the posterior surface of the cornea will affect the accuracy of K measurements and subsequent IOL-power calculations. Little is currently known regarding the accuracy of K measurements in post-DSAEK corneas.

WHAT THIS PAPER ADDS

- For IOL calculations in post-DSAEK eyes, the use of conventional K measurements will not yield a large refractive change. A K measurement obtained using the Pentacam, which accounts for both the anterior and posterior corneal surfaces, is considered the most accurate. This study is the first to assess IOL calculations in post-DSAEK eyes.

## References

[1] Koenig Steven B, Covert Douglas J, Dupps William J, Meisler David M (2007). Visual Acuity, Refractive Error, and Endothelial Cell Density Six Months After Descemet Stripping and Automated Endothelial Keratoplasty (DSAEK). Cornea.

[2] Rao SK, Leung CKS, Cheung CYL (2008). Descemet stripping endothelial keratoplasty: effect of the surgical procedure on corneal optics. Am J Ophthalmol.

[3] Gorovoy MS (2006). Descemet-stripping automated endothelial keratoplasty. Cornea.

[4] Price FW, Price MO (2006). Descemet’s stripping with endothelial keratoplasty in 200 eyes: early challenges and techniques to enhance donor adherence. J Cataract Refract Surg.

[5] Terry MA, Shamie N, Chen ES (2009). Endothelial keratoplasty for Fuchs’ dystrophy with cataract: complications and clinical results with the new triple procedure. Ophthalmology.

[6] Afshari NA, Pittard AB, Siddiqui A (2006). Clinical study of Fuchs cornealendothelial dystrophy leading to penetrating keratoplasty: a 30-yearexperience. Arch Ophthalmol.

[7] Covert DJ, Koenig SB (2007). New triple procedure: Descemet’s stripping and automated endothelial keratoplasty combined with phacoemulsification and intraocular lens implantation. Ophthalmology.

[8] Koenig SB, Covert DJ (2007). Early results of small-incision Descemet's stripping and automated endothelial keratoplasty. Ophthalmology.

[9] Scorcia V, Matteoni S, Scorcia GB (2009). Pentacam assessment of posterior lamellar grafts to explain hyperopization after Descemet's stripping automated endothelial keratoplasty. Ophthalmology.

[10] Prasher P, Muftuoglu O, Bowman RW (2010). Corneal power measurement with a rotating Scheimpflug imaging system after Descemet-stripping automated endothelial keratoplasty. J Cataract Refract Surg.

[11] Clemmensen K, Ivarsen A, Hjortdal J (2015). Changes in Corneal Power After Descemet Stripping Automated Endothelial Keratoplasty. J Refract Surg.

[12] Hong Y, Peng RM, Wang M (2013). Suture pull-through insertion techniques for Descemet stripping automated endothelial keratoplasty in Chinese phakic eyes: outcomes and complications. PLoS One.

[13] Hong Y, Hong J, Xu YG (2013). Comment on phakic descemet stripping automated endothelial keratoplasty: prevalence and prognostic impact of postoperative cataracts. Cornea.

[14] Savini G, Hoffer KJ (2010). Pentacam HR equivalent K-reading. J Refract Surg.

[15] Tang M, Chen A, Li Y (2010). Corneal power measurement with Fourier-domain optical coherence tomography. J Cataract Refract Surg.

[16] Ho JD, Tsai CY, Tsai RJ (2008). Validity of the keratometric index: evaluation by the Pentacam rotating Scheimpflug camera. J Cataract Refract Surg.

[17] Holladay JT, Hill WE, Steinmueller A (2009). Corneal power measurements using scheimpflug imaging in eyes with prior corneal refractive surgery. J Refract Surg.

[18] Hoffer KJ (2000). Clinical results using the Holladay 2 intraocular lens power formula. J Cataract Refract Surg.

[19] Chang SW, Yu CY, Chen DP (2009). Comparison of intraocular lens power calculation by the IOLMaster in phakic and eyes with hydrophobic acrylic lenses. Ophthalmology.

[20] Lee WB, Jacobs DS, Musch DC (2009). Descemet’s stripping endothelial keratoplasty: safety and outcomes. A report by the American Academy of ophthalmology. Ophthalmology.

[21] Holz HA, Meyer JJ, Espandar L (2008). Corneal profile analysis after Descemet stripping endothelial keratoplasty and its relationship to postoperative hyperopic shift. J Cataract Refract Surg.

[22] Bahar I, Kaiserman I, McAllum P (2008). Comparison of posterior lamellar keratoplasty techniques to penetrating keratoplasty. Ophthalmology.

[23] Hua Y, Zhang X, Utheim TP (2016). Evaluation of equivalent keratometry readings obtained by Pentacam HR (high resolution). PLoS One.

[24] Saad E, Shammas MC, Shammas HJ (2013). Scheimpflug corneal power measurements for intraocular lens power calculation in cataract surgery. Am J Ophthalmol.

[25] Symes RJ, Ursell PG (2011). Automated keratometry in routine cataract surgery: comparison of Scheimpflug and conventional values. J Cataract Refract Surg.

[26] Symes RJ, Say MJ, Ursell PG (2010). Scheimpflug keratometry versus conventional automated keratometry in routine cataract surgery. J Cataract Refract Surg.

[27] Shammas HJ, Hoffer KJ, Shammas MC (2009). Scheimpflug photography keratometry readings for routine intraocular lens power calculation. J Cataract Refract Surg.

[28] Gale RP, Saldana M, Johnston RL (2009). Benchmark standards for refractive outcomes after NHS cataract surgery. Eye.

[29] Tang M, Li Y, Avila M (2006). Measuring total corneal power before and after laser in situ keratomileusis with high-speed optical coherence tomography. J Cataract Refract Surg.

[30] Seiler T, McDonnell P (1995). Excimer laser photorefractive keratectomy. Surv Ophthalmol.

[31] Seitz B, Torres F, Langenbacher A (2001). Posterior corneal curvature changes after myopic laser in situ keratomileusis. Ophthalmology.

[32] de Sanctis U, Angeloni M, Zilio C (2011). Corneal power after Descemet stripping automated endothelial keratoplasty using microkeratome-prepared tissues. Opt Vis Sci.

[33] Chaurasia S, Ramappa M, Sangwan V (2012). Cataract surgery after Descemet stripping endothelial keratoplasty. Indian J Ophthalmol.

